# Alternative Splicing: From Abiotic Stress Tolerance to Evolutionary Genomics

**DOI:** 10.3390/ijms24076708

**Published:** 2023-04-04

**Authors:** Bei Gao, Moxian Chen, Melvin J. Oliver

**Affiliations:** 1 State Key Laboratory of Desert and Oasis Ecology, Key Laboratory of Ecological Safety and Sustainable Development in Arid Lands, Xinjiang Institute of Ecology and Geography, Chinese Academy of Sciences, Urumqi 830011, China; 2 Xinjiang Key Laboratory of Conservation and Utilization of Plant Gene Resources, Xinjiang Institute of Ecology and Geography, Chinese Academy of Sciences, Urumqi 830011, China; 3 State Key Laboratory Breeding Base of Green Pesticide and Agricultural Bioengineering, Key Laboratory of Green Pesticide and Agricultural Bioengineering, Ministry of Education, Research and Development Center for Fine Chemicals, Guizhou University, Guiyang 550025, China; 4 Division of Plant Sciences and Interdisciplinary Group, University of Missouri, Columbia, MO 65211, USA

The post-transcriptional regulation of gene expression, in particular alternative splicing (AS) events, substantially contributes to the complexity of eukaryotic transcriptomes and proteomes. The interest in AS events has increased as third-generation sequencing technology allowed researchers to obtain full-length transcripts efficiently and accurately, with or without a reference genome. Research interest surged with the advent of high-fidelity PacBio reads and reduced costs making alternative splicing studies affordable for many laboratories. Therefore, a large body of research has been generated and new areas are being explored. We focused on how variable (or not) the AS control of gene expression is across lineages or within phylogenetically related species with an emphasis on how AS has shaped the evolution of stress responses in plants: an important area which may have positive benefits for agriculture. Agricultural productivity has been threatened by the growing problems associated with climate change; therefore, molecular mechanisms that underpin the response of plants to various kinds of environmental stress have become a major focus for crop improvement.

Thus far, there are a considerable number of reports that have linked AS events to plant stress responses and tolerance [1,2], and this field is growing. It is this burgeoning field of research that provided the inspiration for this Special Issue, entitled “Alternative Splicing: From Abiotic Stress Tolerance to Evolutionary Genomics”, which aims to recruit both original research and review papers that contribute to our understanding of the role of AS in the evolution of plant stress responses and tolerance mechanism. 

The Special Issue contains seven original research articles and three reviews. The species investigated in each of the research articles are phylogenetically and agriculturally diverse; including the moss *Physcomitrium patens*, Arabidopsis, as well as crops such as rice, maize, rapeseed, Tartary buckwheat and *Lycoris longituba* (a Chinese medicinal plant). Most of the research articles concentrate on specific functional genes responsive to abiotic stresses. Notably, functional aspects of genes in plants with agricultural or economic values is a major trend, mirroring the urgent worldwide demand for crops with improved and desired agronomic traits. We are also deeply impressed by the enthusiasm and active participation of the contributing authors to this Special Issue, reflecting the widespread interest in this research topic in the plant sciences. Here, we summarize the contributing articles of this Special Issue.

Deprived nutrient availability can severely impact plant growth and crop yield and led Hua et al. to investigate the GARP transcription factor gene family in allotetraploid rapeseed (*Brassica napus* L.) [3]. They identified a total of 146 members of GARP transcription factor genes in the genome that were phylogenetically classified into five subfamilies. The genomic syntenic relationships within representative eudicot genomes revealed conserved genomic positions for the *BnaGARP* genes. Gene structure, evolutionary selection, *cis*-acting regulatory elements, miRNA-targeting predictions and chromosomal positions for GARP genes were analyzed and displayed. By gleaning insights from transcriptomic data of plants under nutrient stress, Hua et al. focused on *BnaA9.HHO1* and *BnaA1. HHO5* in further functional investigations, suggesting that they possess regulatory roles in response to nutrient stress.

With global warming, heat stress has become a major limiting factor for crop yield improvement. Yang et al. discovered two family members of RNase H1 in the moss *Physcomitrium patens* [4], a representative model for early-diverging land plants. They indicated the presence of homologues to the RNase H1 genes in chlorophytes and charophytes, suggesting an early origin for this gene family. The overexpression of *PpRNH1A* in *P. patens* resulted in OE lines that were more sensitive to heat stress along with a higher number of lipid droplets with less mobility in the transgenic cells. Transcriptomic analyses led the authors to suggest that *PpRNH1A* might be involved in regulating the expression of heat-related genes, such as *DNAJ* and *DNAJC*.

Alternatively, cold environments repress enzyme activities and impact a variety of biological processes. Hou et al. identified 14 histone deacetylase (HDACs) genes from the Tartary buckwheat (*Fagopyrum tataricum*) genome, which were phylogenetically clustered into three subfamilies with distinct tertiary structures [5]. Taking advantage of bioinformatic tools, the authors investigated gene structures, motif compositions, *cis*-acting elements, alternative splicing events, subcellular localizations and gene expression patterns. RNA-Seq read alignments and coverage depth analyses revealed that *FtHDA8-2* was likely subject to an alternative splice event under different low-temperature regimes, suggesting that AS played a role in the response to cold stress. A cold-tolerant variety, Dingku 1, was utilized to illustrate the phenotypic and transcript abundance changes of HDACs under different low temperatures. Overexpression lines, *OE-AtHDA6* and *OE-FtHDA6-1*, were constructed, enabling Hou et al. to demonstrate that *FtHDA6-1* positively regulated cold tolerance.

The membrane attack complex/perforin-like (MACPF) protein superfamily has been extensively studied in animal systems where molecular functions during pathogen infections have been revealed. However, these proteins have been rarely investigated in plants. Zhang et al. focused on the Arabidopsis MACP2 protein and investigated the responsiveness of this protein during both bacterial and fungal pathogen infection [6]. Their report revealed that MACP2 plays a role in promoting pathogen resistance by activating the biosynthesis of tryptophan-derived indole glucosinolates and the salicylic acid signaling pathway. By comparisons of MACP2-OE and -KO lines, indole glucosinolates were revealed to contribute to bacteria resistance in the MACP2-OE plants. Zhang et al., also demonstrated that alternatively spliced *MACP2* isoforms were differentially expressed under pathogen infection, suggesting different roles for AS isoforms, highlighting the prospect that functionally multifaceted expression products can be derived from a single gene.

Two research papers from this Special Issue focused on rice from Professor Ye’s laboratory [7,8], where findings may directly assist in strategies for rice yield or quality improvements. In the first paper, Professor Ye’s team studied the molecular effects of post-anthesis moderate soil drying (MD)n which is proven to be an effective method for promoting starch synthesis and inferior spikelet grain filling. In this paper, the authors probed 1840 AS events in 1392 genes that were differentially expressed between MD and well-water plants. RNA-Seq read alignment depth was used to illustrate the AS events of specific genes of interest. Specifically, the *OsAGPL2* transcript was alternatively spliced to generate transcripts with or without a binding site for the microRNA miR393b, suggesting a potential mechanism for the miRNA-mediated gene regulation of grain filling in inferior spikelets in response to MD. In the second paper, the authors analyzed quantitative trait loci (QTLs) for grain size in rice by developing a suite of F2 populations from a cross of 9311 and CG varieties. Bulked-segregant analysis-seq (BSA-seq) was employed to probe QTLs for various agronomic traits. Within the analysis they detected more than 200 splicing-related loci using whole-genome sequencing, including a splicing-site mutation in the gene *Os03g0841800* (qGL3.3) that generated a truncated open reading frame. Their reported discoveries provide a valuable genetic resource for rice breeding.

Included in the Special Issue is a proteomic and metabolic research paper that focuses on three *Lycoris* species with different alkaloid contents [9], with *Lycoris longituba* exhibiting the highest alkaloid content. This study was included because *Lycoris* bulbs have long been used as traditional Chinese herbal medicine to treat various diseases such as sore throat, abscesses and suppurative wounds. In addition, the SWATH-MS (sequential window acquisition of all theoretical mass spectra)-based quantitative proteomic approach have been widely employed in animal systems but the merits of this technology remain yet to be widely recognized in plants. The comprehensive proteomic analyses presented by the authors were used to identify five candidate proteins encoding enzymes involved in alkaloid biosynthesis, with differential abundance in the three *Lycoris* species. However, particularly fascinating was the discovery of 11 RNA processing-related proteins, which indicated the role of AS in the variation between the three species in alkaloid production.

In addition to seven original research articles, three review papers are included in this Special Issue. Lam et al. summarized the current knowledge on molecular regulations of plant primary and specialized metabolism by alternative splicing [10], highlighting numerous genes that function in plant metabolism that were alternatively spliced at different developmental stages and under stress conditions. They proposed that AS may serve as a fine-tuning regulatory mechanism for plant metabolism. Li et al. concentrated on plant–microbe interactions and discussed the possible mechanisms that connect diverse plant ecotype-phenotype linkages, splicing isoforms, and commensal microbiomes [11]. Jia et al. reviewed the progress in the application of alternative splicing and genome-wide association analyses (GWAS). They proposed that the utilization of GWAS to investigate alternative splicing activity could provide novel insights that could lead to the elucidation of the relationship between AS and the regulation of agronomic traits [12].

In conclusion, as the global climate continues to change and deteriorate, various abiotic stressors and their adverse impacts on crop growth and productivity will present great risk for modern society. The risk of catastrophic crop failures drives the growing research interests in stress biology, especially for crops and plants with economic value. The original research and review articles assembled in the Special Issue provide valuable and novel knowledge of gene function and the potential role of alternative splicing in response to environmental stress. However, despite the well-tailored molecular experimentation for investigating gene function, we noticed the inadequate utilization of bioinformatic methods for comparative and evolutionary genomics to help identify suitable target genes and elucidate their evolutionary trajectories. This gap between “dry lab” and “wet lab” still exists and more collaborative projects need to be facilitated. The published articles in the current Special Issue cannot constitute a comprehensive collection of this interdisciplinary research topic but hopefully provide an impetus to generate more research in this field. With the support of the editorial office, we are delighted to launch a new Special Issue focusing on the same research theme, entitled “Alternative Splicing: From Abiotic Stress Tolerance to Evolutionary Genomics 2.0”, representing our continuous effort on this topic, and we sincerely invite researchers in the field to contribute to this new collection.
